# Hepatitis E virus seroprevalence in patients with HIV in Nizhny Novgorod, Russia

**DOI:** 10.1186/1471-2334-14-S2-P100

**Published:** 2014-05-23

**Authors:** IN Sharipova, AV Berezhnaya, VF Puzyrev, AN Burkov, TI Ulanova

**Affiliations:** 1 RPC “Diagnostic Systems”, Ltd., Nizhny Novgorod, Russia; 2 DSI S.R.L. Saronno, Italy

## Introduction

More than 20 million cases of infection with Hepatitis E (HEV) are registered every year all over the world. By now it is known that chronic Hepatitis E develops in immunosuppressed individuals, including individuals infected with HIV. Studies regarding coinfection of HIV and HEV are limited. Some studies have suggested that patients with HIV may acquire HEV infection more frequently than individuals without HIV. Other studies have not shown differences in HEV prevalence between HIV-infected and non-infected individuals.

## Aim

The aim of this study was to define the degree of prevalence of Hepatitis E markers in the group of HIV infected patients in Nizhny Novgorod. Donor blood serum samples were studies as the control group.

## Materials and methods

505 donor sera provided by Nizhny Novgorod regional blood transfusion station and 500 sera from HIV infected patients provided by Regional Center for prevention and control of AIDS and infection diseases, Nizhny Novgorod, were studied for presence of antibodies to Hepatitis E virus. The levels of IgM or IgG antibodies against HEV were determined with the CE-marked EIA kits (RPC “Diagnostic Systems”, Russia).

## Results

19 (3.8%) of 500 sera from HIV infected patients examined for markers of viral hepatitis E were seropositive. 4 patients out of 500 had both anti-IgM and anti-IgG (0.80%), 5 patients (1.0%) had only anti-IgM and 10 patients had only anti-IgG (2.0%). Detection rate of Hepatitis E antibodies in healthy population of Nizhny Novgorod was 7.3% (37 individuals). Frequency only of IgM marker occurrence was 1.78% (9 individuals); only IgG marker occurred with the frequency 4.75% (24 individuals). Simultaneous presence of IgM and IgG marker in donor sera was 0.79% (4 out of 505 donors).

## Conclusions

According to the results of the study described above, frequency of occurrence of Hepatitis E markers in control group of blood donors was higher than in HIV infected patients. Lower percentage of detection of Hepatitis E markers in HIV-positive patients may be caused by interactive effect of viruses in case of HIV/HEV coinfection.

